# Appropriation of Public Lands for the Indigent Insane

**Published:** 1853

**Authors:** 


### *Appropriation of Public Lands for the Indigent Insane.*

A bill has lately been passed by both Houses of Congress appropriating ten million acres of the public lands to the several States for the benefit of the Indigent Insane. This bill was introduced into Congress and, doubtless, mainly passed, through the instrumentality of Miss Dix, the philanthropist. It is greatly to be regretted that the President has seen proper to veto so humane a bill. We do not profess to be versed in these nice constitutional questions, but we confess we are not able to perceive the distinction between donating the public lands to sane and insane individuals. No one doubts the power of Congress who cede the public domain to the States, or to squatters, who have the shrewdness to make a judicious selection of a home; but it has no power, it seems, to give it to the States for the benefit of the indigent insane. It is a long time since Congress passed so beneficent a bill, and we can scarcely think of one which would have alleviated a larger mass of human suffering, had the President permitted it to become a law.

